# Draft genome of carotenoid-producing endophytic *Pseudomonas* sp. 102515 from *Taxus chinensis*

**DOI:** 10.1128/mra.00139-24

**Published:** 2024-06-28

**Authors:** Ozkan Fidan

**Affiliations:** 1 Department of Bioengineering, Faculty of Life and Natural Sciences, Abdullah Gul University, Kayseri, Turkey; The University of Arizona, Tucson, Arizona, USA

**Keywords:** genome, *Pseudomonas*, endophytic bacteria, *Taxus chinensis*

## Abstract

Here, we report the draft genome sequence of endophytic *Pseudomonas* sp. 102515 isolated from *Taxus chinensis* collected from Logan, UT, USA. The genome is composed of 36 contigs and around 4.9 Mbp in size. The GC content is 66% with an *N*_50_ length of 918.9 kbp and *L*_50_ count of 2.

## ANNOUNCEMENT

Endophytic microbes play significant roles in plant health by providing various benefits such as nitrogen fixation, phosphate solubilization, and growth promotion. Additionally, they are a rich source of bioactive natural products (1). Endophytic *Pseudomonas* sp. 102515 were isolated from the leaves of *Taxus chinensis* and is a high-yield producer of zeaxanthin diglucoside. This isolate can also produce turnerbactin, a siderophore involved in nitrogen fixation. Therefore, *Pseudomonas* sp. 102515 can have plant growth-promoting activity (2). The closest relative strain to this endophyte is *Pseudomonas psychrotolerans*, exhibiting plant growth-promoting activity (3).

The leaves of *T. chinensis* were collected on the Logan campus of Utah State University. The details of isolation and identification were provided in our previous article (2). After the disinfection process, the leaves were ground in an aqueous NaCl solution (0.85%) to release endophytic microorganisms. The tissue extract was diluted and streaked on lysogeny broth (LB) media for the isolation of bacteria. One of the colonies led to the pigment-producing strain, which was identified as *Pseudomonas* sp. 102515 (Taxonomy ID: 3071568).

The endophytic *Pseudomonas* sp. 102515 were grown in LB media from a single colony at 28°C. The genomic DNA was extracted with the EasyPure Genomic DNA Kit (Transgen Biotech). Subsequently, genomic DNA were sent to Refgen Biotech Inc. for sequencing. In the library preparation, the Illumina DNA Prep (Illumina, USA, #20060059) kit was used. During the library preparation, fragmentation, addition of index/barcode sequences, amplification, and purification were carried out by following the manufacturer’s kit instructions. Then, the length distribution of the prepared library was measured using 2100 Bioanalyzer (Agilent, USA, #G2939BA). After quality verification, approximately 5 million paired-end reads with a length of 150 bp were obtained from NovaSeq 6000. 6.24 Gbp of DNA were sequenced, totaling 4,876,136 bp of DNA, which represents 1,279× coverage of the genome. After the sequencing process, the FASTQC (v.1.0.0) tool was used for the quality control of the raw reads. The Trimmomatic (v.0.39) tool was used for cropping operations according to quality values. The Shovill (v.1.1.0) tool and SPAdes assembler (v.3.9.0) were used in *de novo* assembly processes (4, 5).

The genome is composed of 36 contigs and around 4.9 Mbp in size. The GC content is 66% with an *N*_50_ length of 918.9 kbp and *L*_50_ count of 2. The NCBI Prokaryotic Genome Annotation Pipeline (PGAP) was used for annotation (6). Based on the PGAP (v.4.9), the genome includes 4,575 genes and 4,444 protein-coding sequences. CheckM analysis (v.1.2.2) was used to calculate the completeness of the genome, which was found as 98.56%, while 0.61% contamination was reported (7). Comprehensive genome analysis was performed using the Bacterial and Viral Bioinformatics Resource Center (BV-BRC) (v.3.35.5) (8). The annotation RAST tool kit (RASTtk) (v.1.073) had 900 hypothetical proteins and 3,615 proteins with functional assignments (9). The proteins with functional assignments included 1,136 proteins with Enzyme Commission numbers (10), 973 with Gene Ontology assignments (11), and 862 proteins that were mapped to KEGG pathways (12). According to BV-BRC annotation, this genome had 4,287 proteins that belong to the genus-specific protein families (PLFams) and 4,347 proteins that belong to the cross-genus protein families (PGFams) (13).

## Data Availability

The Illumina raw reads were uploaded to the SRA database under the accession number of SRX24173677. The generated contigs were uploaded on NCBI under Biosample ID SAMN37118173. Two contigs smaller than 200 bp were discarded during the Genbank upload. The Whole Genome Shotgun project has been deposited at DDBJ/ENA/GenBank under accession JAVHTY000000000. The version described in this paper is version JAVHTY010000000.1.
